# UNDERSTANDING SEX DIFFERENCES IN OLDER PERSONS WITH NEURODEGENERATIVE DISEASES

**DOI:** 10.1093/geroni/igz038.059

**Published:** 2019-11-08

**Authors:** Michael A Campitelli, Laura C Maclagan, Christina Diong, Longdi Fu, Amy Y Yu, Lorne Zinman, Rick Swartz, Susan E Bronskill

**Affiliations:** 1 Institute for Clinical Evaluative Sciences (ICES), Toronto, Ontario, Canada; 2 ICES, Toronto, Ontario, Canada; 3 Sunnybrook Health Sciences Centre, Toronto, Ontario, Canada

## Abstract

Sex differences in the incidence, prevalence, and clinical presentation of neurodegenerative diseases have been previously documented, however, sex differences in how individuals with neurodegenerative diseases access the health system remain understudied. Clinical trials and observational studies often do not report data stratified by sex, which limits the understanding of sex-related differences in persons with neurodegenerative diseases. This session will highlight both opportunities and methodological challenges researchers face when undertaking sex and gender research in persons with neurodegenerative diseases using two case studies: 1) sex differences in health service utilization prior to a diagnosis of Amyotrophic Lateral Sclerosis (ALS); and 2) sex differences in care needs and survival among persons who are admitted to a nursing home after a stroke. The findings of these studies may support the development of guidelines and care plans to meet the needs of men and women with neurodegenerative disorders in varied care settings.

